# Relationships Between Traditional Chinese Medicine Constitution and Age-Related Cognitive Decline in Chinese Centenarians

**DOI:** 10.3389/fnagi.2022.870442

**Published:** 2022-05-09

**Authors:** Zhigao Sun, Ping Ping, Yulong Li, Long Feng, Fan Liu, Yali Zhao, Yao Yao, Pei Zhang, Shihui Fu

**Affiliations:** ^1^Department of Traditional Chinese Medicine, Hainan Hospital of Chinese People’s Liberation Army General Hospital, Sanya, China; ^2^Main Station of Drug Instrument Supervision and Inspection, Chinese People’s Liberation Army Joint Logistic Support Force, Beijing, China; ^3^Department of Geriatric Cardiology, Chinese People’s Liberation Army General Hospital, Beijing, China; ^4^Department of Anesthesiology, Hainan Hospital of Chinese People’s Liberation Army General Hospital, Sanya, China; ^5^Central Laboratory, Hainan Hospital of Chinese People’s Liberation Army General Hospital, Sanya, China; ^6^Geriatrics Division, Center for the Study of Aging and Human Development, Duke University School of Medicine, Durham, NC, United States; ^7^Center for Healthy Aging and Development Studies, National School of Development, Peking University, Beijing, China; ^8^School of Life Sciences, Beijing Institute of Technology, Beijing, China; ^9^Department of Cardiology, Hainan Hospital of Chinese People’s Liberation Army General Hospital, Sanya, China

**Keywords:** age-related cognitive decline, Chinese centenarians, Mini-Mental State Examination, Traditional Chinese Medicine constitution, Qi depression

## Abstract

**Background:**

Age-related cognitive decline (ARCD) is a common condition among older adults, affecting 100 million people worldwide. Traditional Chinese Medicine’s (TCM) constitution is closely related to the occurrence and development of diseases in the elderly population. However, little is known about the relationships between TCM constitution and ARCD in the super-aged population. The present study aimed to investigate the relationships between the TCM constitution and ARCD in Chinese centenarians and to explore the application of the constitution to prevent ARCD in the elderly population.

**Methods:**

Each participant underwent a standardized epidemiological investigation and physical examination, based on the China Hainan Centenarian Cohort Study. Data on the demographic characteristics and TCM constitution were collected using structured questionnaires.

**Results:**

The present study included 636 centenarians aged 100–116 years. The prevalence of ARCD was 87.7% (*n* = 558 centenarians). In multiple linear regression analysis, an inverse relationship between Qi depression and Mini-Mental State Examination scales was significant after controlling for a wide range of other factors (*P* < 0.05). In multiple logistic regression analysis, Qi depression was positively associated with ARCD after full adjustment (*P* < 0.05).

**Conclusion:**

As the first study in the world, the present study provides strong epidemiological evidence that Qi depression has a significant relationship with ARCD in Chinese centenarians, and regulating Qi depression may be a valuable method to prevent and treat ARCD in the elderly population.

## Introduction

Age-related cognitive decline (ARCD) is a progressive neurodegenerative disease that is more prevalent in oldest-old adults than in younger adults (Kioussis et al., 2021; Liu et al., 2021). According to data from the WHO in 2017, approximately 100 million people have ARCD, and this number will further increase with the increased growth of global aging (Carman et al., 2014). ARCD has created a serious social and economic burden on society. ARCD has been clinically proven to be the prodromal stage of dementia and preventing the development of ARCD has become a popular research topic in the medical field (Gorelick et al., 2016). Unfortunately, current drugs, such as acetylcholinesterase inhibitors and non-competitive NMDA receptor modulators, are still controversial for improving cognitive function (Meng et al., 2017).

Traditional Chinese Medicine (TCM) constitution has been proposed by the academician Wang Qi and is widely used in the prevention of aging diseases (Wang, 1995). It includes Qi deficiency, Yang deficiency, Yin deficiency, Qi depression, Damp-heat, Phlegm-dampness, Blood-stasis, Idiosyncrasy, and Pinghe (Li et al., 2019). Based on the theories, the aging of the human body is marked by the gradual depression of Qi. Qi depression results in the congestion of blood circulation and accumulation of pathological products and affects learning and memory functions by inhibiting the expression of central neurotransmitters (Chiang et al., 2014; De Oliveira et al., 2014).

Extensive studies have shown that Chinese herbal compound prescriptions for regulating Qi depression can improve the forgetfulness and unresponsiveness of ARCD, such as Chaihu Shugan San (Zeng et al., 2019) and Kai Xin San (Dang et al., 2009). The mechanisms related to neuroprotection include decreasing β-amyloid precursor protein processing, inhibiting Aβ plaques, reducing oxidative stress, modulating immune function, and reducing apoptotic proteins (Lu et al., 2017; Hu et al., 2018; Liu et al., 2019). However, epidemiological research is lacking, especially in Chinese centenarians. The China Hainan Centenarian Cohort Study (CHCCS) had a considerable sample size and provided credible research opportunities. Therefore, the present study aimed to investigate the relationships between the TCM constitution and ARCD and to explore the application of the constitution to prevent ARCD in the elderly population.

## Materials and Methods

### Ethics Statements

The study protocol was approved by the Ethics Committee of the Hainan Hospital of Chinese People’s Liberation Army General Hospital (301hn11201601). Informed consent was obtained from all the participants and their legal guardians.

### Study Population

The CHCCS, one of the largest centenarian health interdisciplinary studies, was conducted in the Hainan Province, where there is a longevity area with the highest density of centenarians in China. The CHCCS enrolled 910 centenarians older than 100 years of age between July 2014 and December 2016 from 18 cities and counties in the Hainan Province, China. The ages of the centenarians were provided and verified by the Hainan Civil Affairs Bureau. Based on a standardized protocol, in-person interviews, physical examinations, and blood analyses were conducted by a well-trained research team at the Chinese People’s Liberation Army General Hospital *via* home visits. This interdisciplinary research team included TCM physicians, internists, geriatricians, cardiologists, endocrinologists, nephrologists, and nurses. The research design and survey methodology have been previously described (He et al., 2018; Fu et al., 2021a,b). In summary, there were 636 centenarians with the complete data included in the final analysis.

### Cognitive Function

Cognitive function was measured using the Mini-Mental State Examination (MMSE) scale with high sensitivity and specificity (Fu et al., 2018). The scale includes 30 items (1 point for correct answer, 0 point for incorrect answer, or no knowledge of the answer), including orientation, memory, attention, calculation, language, and spatial ability. The total score of the scale ranges from 0 to 30 points, and the standards of ARCD are as follows: illiteracy ≤17 points, elementary school level ≤20 points, middle school level ≤22 points, and university level ≤23 points (Creavin et al., 2016).

### Traditional Chinese Medicine Constitution

Traditional Chinese Medicine constitution was assessed based on the simplified version of the academician Wang Qi’s TCM constitution questionnaire for the elderly population in combination with the actual situation in the CHCCS (Xuan, 2013; Zhu et al., 2018). This scale includes 24 pentad items, and each item scored from 1 to 5 points, covering eight constitutions, including Qi deficiency (3 items, 15 points in total), Yang deficiency (4 items, 20 points in total), Yin deficiency (4 items, 20 points in total), Qi depression (2 items, 10 points in total), Damp heat (3 items, 15 points in total), Phlegm dampness (1 item, 5 points in total), Blood stasis (3 items, 15 points in total), and Idiosyncrasy (4 items, 20 points in total). Each item in the questionnaire corresponds to a constitution, and the score is proportional to the prevalence of TCM constitution.

### Concomitant Variables

Demographic characteristics, including age, sex, sleep duration (at night), education level (illiterate or above), fish intake (every day), fruit intake (more than three times a week), outdoor walking (more than 1 h a day), living with family, visual impairment (unable to live normally), auditory impairment (unable to live normally), and constipation (more than 2 days of defecation interval), were collected by strictly trained investigators using structured questionnaires and standardized procedures.

Height (H) and weight (W) were measured using a standardized scale (SeCa GmbH, Müllheim, Germany).

Body mass index (BMI) = W/H^2^

Systolic blood pressure (SBP) and diastolic blood pressure (DBP) were measured twice using a sphygmomanometer (Omron Hem-7200, Kyoto, Japan), with an average of two measurements used.

Blood samples of centenarians were collected by professional nurses and transported (4°C) to the central laboratory of our hospital within 4 h. Hemoglobin (Hb) levels were measured using a blood autoanalyzer (SYSMEX XS-800I, Hyogo, Japan). Plasma triglyceride (TG), total cholesterol (TC), fasting blood glucose (FBG), and albumin (ALB) levels were determined by enzyme colorimetry (Roche Products Ltd., Basel, Switzerland) using a fully automatic biochemical autoanalyzer (COBAS c702; Roche Products Ltd., Basel, Switzerland).

### Statistical Analysis

Continuous variables with a normal distribution are described as mean ± *SD*, and those with a skewed distribution are described as median (interquartile range). Categorical variables are presented as percentages.

The *t*-test (mean ± *SD*) and the *U*-test (median) were used to compare the two types of continuous variables, and the chi-square test was used to compare categorical variables. Multiple logistic regression analysis was used to assess the relationships between TCM constitution and ARCD as a categorical variable, and multiple linear regression analysis was used to analyze the relationships between TCM constitution and MMSE scales as a continuous variable. Adjustments were made for age, sex, sleep duration, education level, fish intake, fruit intake, outdoor walking, social communication, visual impairment, auditory impairment, BMI, SBP, DBP, Hb, ALB, TG, TC, and FBG levels, which were significantly different between participants with and without ARCD (*P* < 0.05) in the *t*-test, *U*-test, or the chi-square test.

Statistical analyses were performed using SPSS 17 software package (IBM Corp., Armonk, NY, United States). Statistical significance was set at *P* < 0.05.

## Results

**FIGURE 1:**
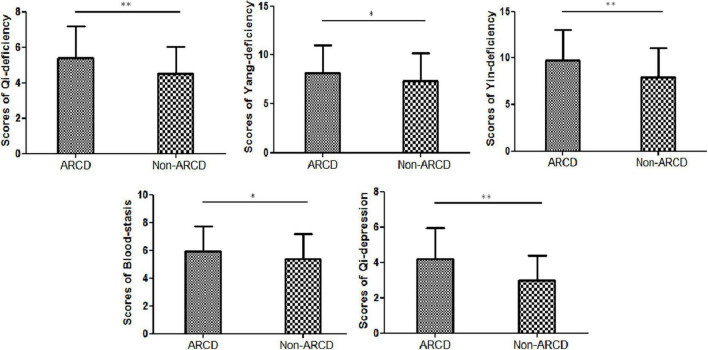
Traditional Chinese Medicine (TCM) constitution of centenarians with and without age-related cognitive decline (ARCD). **p < 0.01; *p < 0.05.

The present study included 636 centenarians aged 100–116 years, including 520 women and 116 men. The proportion of centenarians with ARCD was 87.7% (*n* = 558). As shown in Table 1, the scores of Qi deficiency, Yang deficiency, Yin deficiency, Qi depression, and Blood-stasis were significantly higher in centenarians with ARCD than in those without ARCD (all, *P* < 0.05; Figure 1). Older age; female sex; higher proportions of illiterate individuals, individuals living with family, and individuals with constipation; and higher TG levels were more prominent among centenarians with ARCD than among those without ARCD (all, *P* < 0.05; Table 1). Centenarians with ARCD had a lower proportion of outdoor walking and lower levels of ALB and Hb than those without ARCD (*P* < 0.05; Table 1).

**TABLE 1:** Characteristics of all participants with and without age-related cognitive decline (ARCD).

Characteristics	Total sample (*n* = 636)	ARCD (*n* = 558)	Non-ARCD (*n* = 78)	*P*
Qi-deficiency*	5.27 ± 1.81	5.38 ± 1.82	4.51 ± 1.50	<0.001
Yang-deficiency*	8.04 ± 2.85	8.14 ± 2.83	7.33 ± 2.88	0.021
Yin-deficiency*	9.54 ± 3.29	9.75 ± 3.26	7.90 ± 3.14	<0.001
Phlegm-dampness*	1.92 ± 0.82	1.92 ± 0.80	1.94 ± 0.95	0.81
Damp-heat*	5.02 ± 1.63	5.04 ± 1.59	4.87 ± 1.88	0.38
Blood-stasis*	5.86 ± 1.82	5.92 ± 1.82	5.39 ± 1.80	0.02
Qi-depression*	4.04 ± 1.76	4.18 ± 1.76	3.00 ± 1.39	<0.001
Idiosyncrasy*	5.75 ± 1.80	5.77 ± 1.75	5.61 ± 2.14	0.50
Age, year*	102.75 ± 2.7	102.85 ± 2.80	102.02 ± 2.13	0.012
Females, %	520 (81.7)	469 (84.05)	51 (65.38)	<0.001
Sleep duration, h*	8.07 ± 2.55	8.05 ± 2.51	8.20 ± 2.82	0.633
Illiterate, %	571 (89.7)	510 (91.3)	61 (78.20)	<0.001
Living with the family, %	555 (86.3)	493 (88.3)	62 (79.48)	0.028
Fish intake, %	208 (32.7)	179 (32.07)	29 (37.17)	0.368
Fruit intake, %	98 (15.4)	83 (14.87)	15 (19.23)	0.318
Outdoor walking >1 h/d, %	222 (34.9)	177 (31.72)	45 (57.69)	<0.001
Visual impairment, %	148 (23.2)	136 (24.37)	12 (15.38)	0.078
Auditory impairment, %	165 (25.9)	149 (26.70)	16 (20.51)	0.243
Constipation, %	148 (23.2)	140 (25.08)	8 (10.25)	0.004
BMI, kg/m^2^*	18.27 ± 3.71	18.21 ± 3.81	18.64 ± 3.05	0.365
SBP, mmHg*	152.91 ± 25.03	152.73 ± 24.92	154.21 ± 26.00	0.643
DBP, mmHg*	75.62 ± 13.19	75.53 ± 13.17	76.30 ± 13.40	0.649
TG, mmol/L*	1.18 ± 0.63	1.20 ± 0.66	1.04 ± 0.39	0.04
TC, mmol/L*	4.67 ± 0.99	4.65 ± 0.97	4.82 ± 1.14	0.168
FBG, nmol/L*	5.14 ± 1.47	5.16 ± 1.50	5.00 ± 1.24	0.427
ALB, g/L*	38.49 ± 3.96	38.34 ± 4.01	39.60 ± 3.42	0.011
Hb, g/L*	112.83 ± 16.74	112.22 ± 16.97	117.25 ± 14.35	0.015

*Mean ± standard deviation. ARCD, age-related cognitive decline; BMI, body mass index; SBP, systolic blood pressure; DBP, diastolic blood pressure; TG, triglyceride; TC, total cholesterol; FBG, fasting blood glucose; ALB, albumin; Hb, hemoglobin.

In multiple linear regression analysis, Qi depression, age, proportions of illiterate individuals and individuals living with family, and TG levels were inversely associated with MMSE scales, whereas the proportion of outdoor walking and ALB levels were positively associated with MMSE scales after controlling for a wide range of other factors (all, *P* < 0.05; Table 2). In multiple logistic regression analysis, Qi depression, proportions of individuals with constipation and individuals living with family, and TG levels were positively associated with MMSE scales, whereas the proportion of outdoor walking was inversely associated with MMSE scales after full adjustment (*P* < 0.05; Table 3).

**TABLE 2:** Multiple linear regression analysis between Traditional Chinese Medicine (TCM) constitution and Mini-Mental State Examination (MMSE) scales.

Variables	Standardized beta	95% CI	*P*
Qi-deficiency	−0.080	−0.566 ∼−0.009	0.058
Yang-deficiency	0.000	−0.186 ∼ 0.184	0.990
Yin-deficiency	−0.053	−0.295 ∼ 0.094	0.310
Qi-depression	−0.206	−1.048 ∼−0.423	<0.001
Blood-stasis	−0.031	−0.430 ∼ 0.214	0.510
Age	−0.077	−0.343 ∼−0.006	0.043
Females	−0.067	−2.493 ∼ 0.293	0.122
Illiterate	−0.148	−4.707 ∼−1.333	<0.001
Living with the family	−0.127	−3.836 ∼−0.977	0.001
Outdoor walking	0.167	1.184 ∼ 3.206	<0.001
Constipation	−0.074	−2.194 ∼−0.010	0.052
TG	−0.130	−2.011 ∼−0.514	0.001
ALB	0.139	0.089 ∼ 0.354	0.001
Hb	0.019	−0.024 ∼ 0.038	0.650

*Adjusted for Qi-deficiency, Yang-deficiency, Yin-deficiency, Qi-depression, Blood-stasis, age, females, illiterate, living with the family, outdoor walking, constipation, TG, ALB, and Hb (P < 0.05 in Table 1). TCM, Traditional Chinese Medicine; MMSE, Mini-Mental State Examination; CI, confidence interval; TG, triglyceride; ALB, albumin; Hb, hemoglobin.*

**TABLE 3:** Multiple logistic regression analysis between TCM constitution and ARCD.

Variables	OR	95% CI	*P*
Qi-deficiency	1.167	0.943 ∼ 1.445	0.156
Yang-deficiency	1.032	0.896 ∼ 1.188	0.660
Yin-deficiency	1.023	0.892 ∼ 1.174	0.744
Qi-depression	1.521	1.162 ∼ 1.989	0.002
Blood-stasis	1.171	0.928 ∼ 1.479	0.184
Age	1.122	0.980 ∼ 1.284	0.095
Females	1.662	0.748 ∼ 3.693	0.212
Illiterate	1.752	0.685 ∼ 4.482	0.242
Living with the family	0.353	1.246 ∼ 6.426	0.013
Outdoor walking	0.319	0.167 ∼ 0.612	0.001
Constipation	5.112	1.760 ∼ 14.845	0.003
TG	2.841	1.324 ∼ 6.098	0.007
ALB	0.953	0.869 ∼ 1.045	0.308
Hb	0.985	0.964 ∼ 1.006	0.154

*Adjusted for Qi-deficiency, Yang-deficiency, Yin-deficiency, Qi-depression, Blood-stasis, age, females, illiterate, living with the family, outdoor walking, constipation, TG, ALB, and Hb (P < 0.05 in Table 1). TCM, Traditional Chinese Medicine; ARCD, age-related cognitive decline; OR, odds ratio; CI, confidence interval; TG, triglyceride; ALB, albumin; Hb, hemoglobin.*

## Discussion

As the first study in the world, we found that Qi depression was inversely associated with MMSE scales and positively associated with ARCD in Chinese centenarians. These associations were significant after adjusting for demographic characteristics and other potential confounders.

Dysfunction of memory, language, reaction, and understanding are the main clinical manifestations of dementia, which is highly consistent with Qi depression in TCM (Byers and Yaffe, 2011; Liu et al., 2012). A study (Zhang et al., 2021) in Macau, including 313 elderly people (age, 77.10 ± 8.23 years), showed that Qi depression was negatively correlated with neurocognitive scores (β = −2.66, 95% CI: −4.99 to −0.33), and it was mainly manifested in affecting vision dimensions of space capability. This finding indicates that Qi depression is closely related to ARCD, which is consistent with our study’s results. Positron emission tomography showed that cerebral oxygen metabolism in patients with Qi depression was significantly reduced, and this reduction was also related to the severity of dementia (Yu, 2006). Animal experiments have also shown that in Qi depression model rats, cell peroxidation was enhanced, and free radicals were increased, presenting as abnormal test results of the water maze and decreased cognition and learning ability (Zhu et al., 2019). In addition, previous studies have shown that traditional antidepressant prescriptions, such as Xiaoyao Powder and Chaihu Shugan Powder, could significantly increase MMSE scales and improve overall cognition (Wu, 2015; Liu et al., 2018). This mechanism is related to increased acetyl cholinesterase (AChE) content and reduced choline acetyltransferase (ChAT) expression (Wang et al., 2014), suggesting that with the development of the biological-psychological-social medical model, Qi depression caused by an accumulation of negative emotions has been proven to be closely related to the progression of cognitive decline (Zhou et al., 2006; Ismail et al., 2018; Zhang et al., 2021).

Based on the present study with relatively large sample size, it has important clinical significance for the prevention of ARCD by regulating TCM constitution in the elderly population, which is also an advantage of TCM. In the future, research can be conducted to slow down and alleviate cognitive impairment in the elderly population by regulating Qi.

While highlighting the results of this study, some limitations should be acknowledged. First, this study was based on a cross-section, hence causality could not be inferred. Second, the lack of clear biological markers for Qi depression is a difficult point in the study of the relationships between TCM constitution and ARCD.

## Conclusion

The present study provides strong epidemiological evidence that Qi depression has a significant relationship with ARCD in Chinese centenarians and regulating Qi depression may be a valuable method to prevent and treat ARCD in the elderly population.

## Data Availability Statement

The raw data supporting the conclusions of this article will be made available by the authors, without undue reservation.

## Ethics Statement

The studies involving human participants were reviewed and approved by the Ethics Committee of Hainan Hospital of Chinese People’s Liberation Army General Hospital (301hn11201601). The patients/participants provided their written informed consent to participate in this study.

## Author Contributions

YZ, YY, and SF designed the study and performed the investigation. ZS, PP, YL, and SF conducted the statistical analyses and drafted the manuscript. PZ, FL, and YZ revised the manuscript. All authors have read and approved the final manuscript.

## Conflict of Interest

The authors declare that the research was conducted in the absence of any commercial or financial relationships that could be construed as a potential conflict of interest.

## Publisher’s Note

All claims expressed in this article are solely those of the authors and do not necessarily represent those of their affiliated organizations, or those of the publisher, the editors and the reviewers. Any product that may be evaluated in this article, or claim that may be made by its manufacturer, is not guaranteed or endorsed by the publisher.
